# A case of human seminal plasma anaphylaxis masked by *Anisakis* sensitization: Successful pregnancy via *in vitro* fertilization

**DOI:** 10.1016/j.jacig.2026.100768

**Published:** 2026-07-29

**Authors:** Makoto Nojo, Shogo Nishii, Shintaro Suzuki, Tomoki Uno, Kaoru Mochizuki, Yoshito Miyata, Yoshio Watanabe, Sojiro Kusumoto, Akihiko Sekizawa, Hironori Sagara, Akihiko Tanaka

**Affiliations:** a Division of Respiratory Medicine and Allergology, Department of Medicine, Showa Medical University School of Medicine, Tokyo, Japan; b Department of Obstetrics and Gynecology, Showa Medical University School of Medicine, Tokyo, Japan; c Department of Medical Education, Showa Medical University School of Medicine, Tokyo, Japan

**Keywords:** Anaphylaxis, *Anisakis*, basophil activation test, delayed anaphylaxis, fertilization *in vitro*, human seminal plasma allergy, immunologic tests, infertility, reproductive techniques, skin tests

## Abstract

In refractory nocturnal anaphylaxis, hidden allergens such as human seminal plasma allergy should be considered. Basophil activation testing using simple semen preparations may provide supportive *ex vivo* evidence, and *in vitro* fertilization provides a definitive, allergen-free route to successful pregnancy.

Human seminal plasma allergy (HSPA) is a rare IgE-mediated disorder triggered by exposure to proteins in a partner’s semen.^1^ Although only about 80 cases have been documented, its true prevalence is likely higher owing to underrecognition, with extrapolations suggesting up to 40,000 potential cases in the United States.^2^ Diagnosis is often missed, as patients may hesitate to report sexual details and clinicians may not inquire systematically. The challenge is compounded in the 30% of cases presenting atypically with only systemic or delayed reactions and lacking classic localized symptoms.^3^ The key historical clue is the complete prevention of symptoms with barrier protection. In Japan, *Anisakis* simplex is a common allergen, accounting for 10% to 20% of adult anaphylaxis, which can divert attention from other causes.^4^ We present the case of a 35-year-old woman with recurrent nocturnal anaphylaxis that was initially attributed to high levels of *Anisakis*-specific IgE (sIgE). Sexual history and functional testing revealed HSPA; *in vitro* fertilization (IVF) avoided exposure to seminal plasma and enabled healthy childbirth. To our knowledge, this is the first full case report of IVF-enabled live birth in a patient with HSPA.

## Case presentation

A 35-year-old woman with a history of atopic dermatitis and sensitization to house dust mite and rabbit dander, as well as an extensive history of pet ownership, presented with a 3-year history of recurrent, nocturnal anaphylaxis, with more than 10 episodes. Episodes, characterized by generalized urticaria, facial angioedema, dyspnea, and abdominal pain, occurred 1 to 3 hours after she had gone to bed and required emergency treatment with intramuscular epinephrine (0.3 mg). The initial investigations were unrevealing for common food triggers. Because serum testing showed a markedly elevated level of *Anisakis* sIgE (51.50 kU/L [class 5]), *Anisakis*-induced delayed anaphylaxis was initially suspected. However, the absence of raw seafood ingestion within the preceding 7 days and the persistence of attacks despite strict seafood avoidance weakened a causal role for *Anisakis*.

Systematic reevaluation uncovered a key pattern: all of the patient’s anaphylactic episodes consistently occurred within 60 minutes after condomless intercourse, whereas none occurred with condom use. This perfect correlation provided a stronger causal link than the discordant *Anisakis* sIgE level. These findings prompted a diagnostic shift toward human seminal plasma allergy, which was supported by the patient’s history and the clear preventive effect of barrier protection.

**Fig 1:**
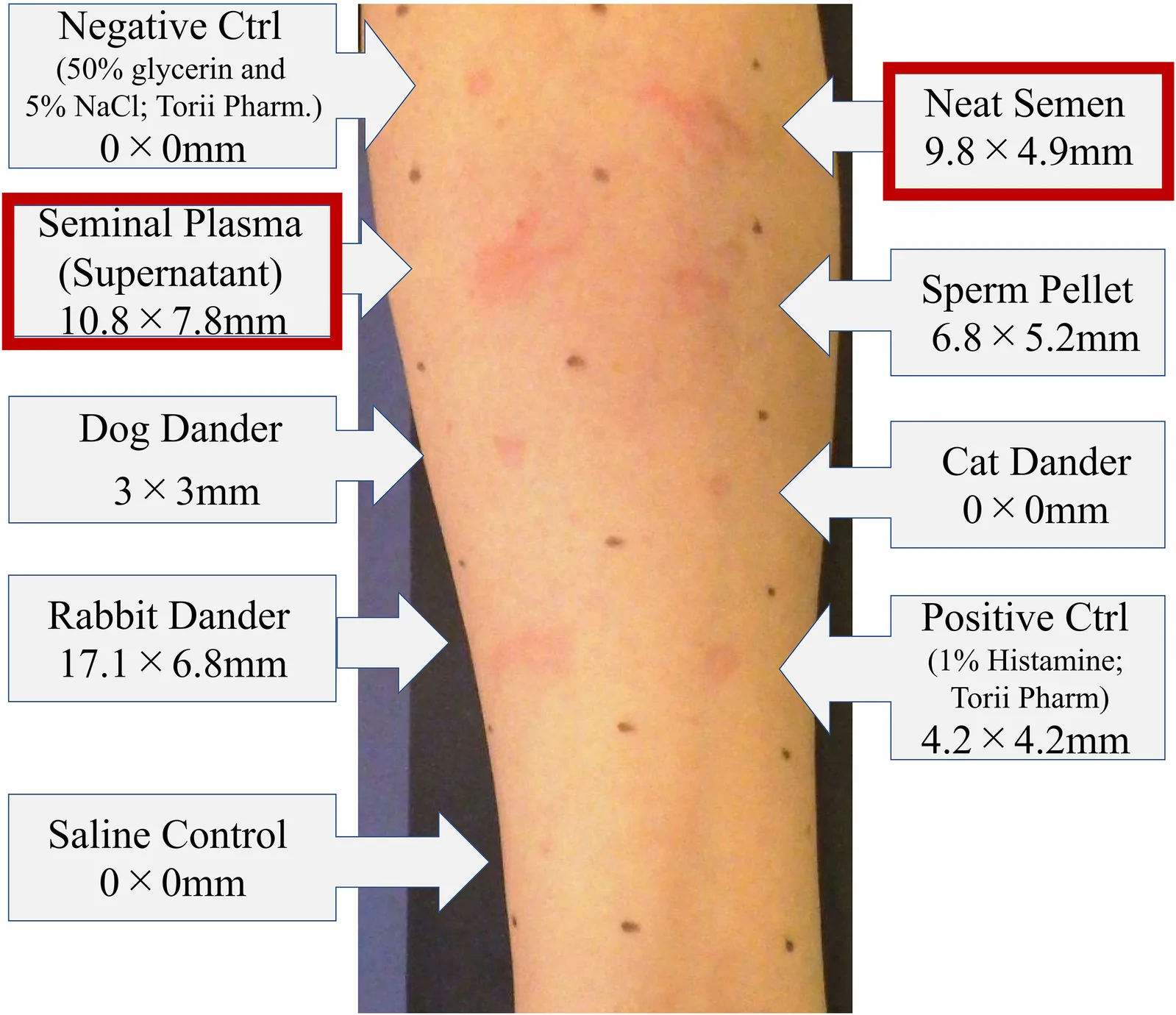
SPT with partner-derived semen preparations confirming human seminal plasma allergy. With consent, freshly ejaculated semen was allowed to liquefy at ambient temperature for approximately 30 minutes, diluted 1:1 with saline, and centrifuged at 4°C at 1000 rpm / 181 × *g* for 30 minutes to obtain seminal plasma supernatant and sperm pellet. SPT was performed using bifurcated needles. Reactions are reported in terms of wheal size only, expressed as the longest diameter times the perpendicular diameter in millimeters; flare responses were not measured or reported. SPT showed positive wheal responses to semen-derived samples, including reactions to neat semen, seminal plasma supernatant, and sperm pellet, thus supporting the diagnosis of human seminal plasma allergy. Reference dander SPT showed sensitization to dog and rabbit dander. The photograph was obtained 30 minutes after testing; therefore, the histamine control reaction may appear smaller than at its expected peak.

**Fig 2:**
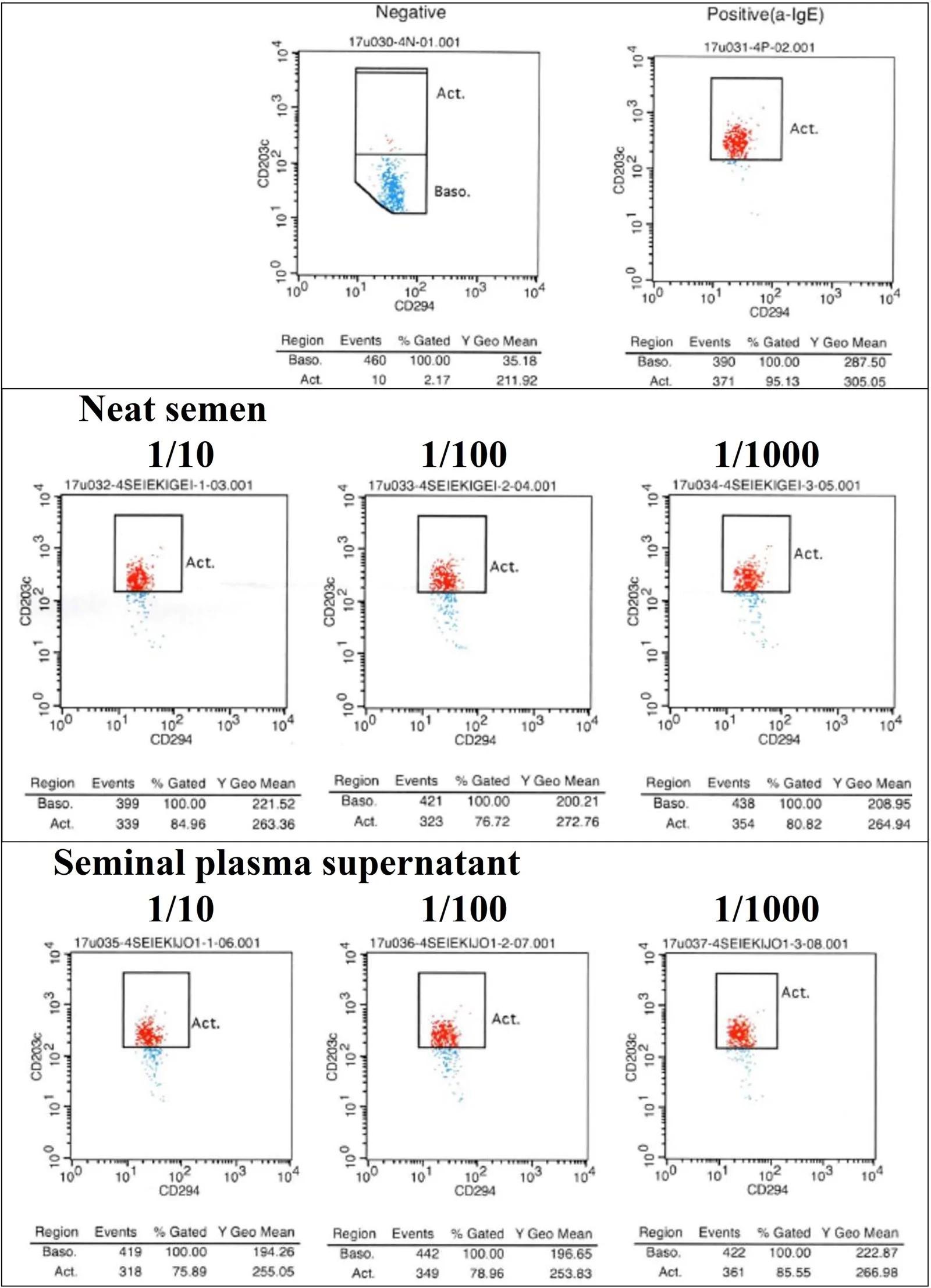
BAT with serial dilutions of neat semen and seminal plasma supernatant obtained from the patient’s partner. The patient’s basophils were stimulated with neat semen and seminal plasma supernatant at dilutions of 1:10, 1:100, and 1:1000; CD203c expression was analyzed by flow cytometry. The tested preparations induced robust basophil activation, with a peak response of 85.6%. The reaction demonstrated by the negative control was minimal, and the anti-FcεRI positive control showed near-maximal activation. These findings indicate that neat semen can elicit comparable basophil activation without prior protein fractionation.

To investigate suspected HSPA, skin prick testing (SPT) and basophil activation testing (BAT) using partner-derived semen preparations were performed. Fresh ejaculate was obtained from the patient’s partner in the outpatient setting. After microscopic confirmation of spermatozoa, the sample was allowed to liquefy at room temperature for approximately 30 minutes, diluted 1:1 with sterile saline, and centrifuged at 4°C (1000 rpm / 181 × *g* for 30 minutes) to separate the seminal plasma (supernatant) from the sperm pellet by using a low-speed, short-spin protocol without specialized equipment. BAT was performed by a commercial laboratory (BML, Japan). The results of SPT in response to the partner-derived semen preparations were positive (Fig 1). BAT demonstrated robust activation of the patient's basophils with neat semen (up to 85.6% activation; not only seminal plasma), supporting functional IgE-mediated reactivity (Fig 2).

On the basis of the patient’s history and functional testing, the diagnosis was revised to HSPA. *Anisakis*-induced anaphylaxis was considered unlikely for the index episodes; after stepwise reintroduction to seafood (in the form of sashimi), no recurrence occurred during the next 3 years.^5^ Although future *Anisakis*-related reactions could not be excluded, strict condom use led to complete resolution of the patient’s symptoms. Considering her age (35 years), strong desire for early conception, and need to minimize allergic risk, the couple chose IVF over intrauterine insemination (IUI) to fully avoid seminal plasma exposure. IVF was performed twice, resulting in an uncomplicated pregnancy and the birth of a healthy infant. In this context, IVF functioned not only as a fertility treatment but also as a safe and preventive strategy tailored to the patient’s condition.

Written informed consent for publication, including clinical data and images, was obtained from the patient.

## Discussion

This case illustrates diagnostic inertia: anchoring of causality to sIgE despite poor exposure concordance. Although the patient’s level of *Anisakis* sIgE was high, prior *Anisakis* infection or house dust mite cross-reactivity could have caused persistent sensitization^4^^,^^5^; therefore, sIgE alone did not establish causality for the aforementioned episodes. The episodes lacked exposure to raw seafood within 7 days, persisted despite avoidance, and stopped with condom use; moreover, sashimi reintroduction caused no recurrence for 3 years. Thus, *Anisakis* was an unlikely explanation for the index episodes, although future *Anisakis* reactions cannot be excluded. This outcome should not be generalized to unrestricted raw fish intake; however, despite global recommendations for universal freezing of fresh fish, in Japan, where anisakiasis is common, allergic manifestations occur in only approximately 3% of cases, and consumption of nonfrozen raw fish is culturally frequent, *Anisakis* sIgE positivity alone should not mandate blanket avoidance, and individualized risk counseling is required.^5^ HSPA is notoriously underdiagnosed, partly because as in our case, up to 30% of cases, lack local genital symptoms and present with delayed systemic reactions, mimicking food-induced anaphylaxis.^1^, ^2^, ^3^ This underscores the fact that for recurrent, unexplained anaphylaxis (particularly with a nocturnal pattern), a systematic sexual history should be considered a mandatory component of the initial workup, on a par with dietary history.

In the absence of commercial extracts for HSPA, functional assays are indispensable. SPT using the partner's seminal plasma provides direct *in vivo* evidence of mast cell reactivity.^1^ For a patient with a history of anaphylaxis, a formal challenge (ie, unprotected intercourse) is ethically prohibitive. In this context, BAT provided a safe *ex vivo* functional test complementing history taking and SPT. Although not standardized for HSPA, concordant activation in response to neat semen and seminal plasma supported IgE-mediated reactivity without *in vivo* exposure.^6^ Together, the patient’s clinical history, SPT results, and BAT results supported a diagnostic shift from *Anisakis*-induced anaphylaxis to HSPA.

The central contribution of this case is support for IVF-enabled live birth in patients with severe HSPA. For pregnancy, use of desensitization and use of assisted reproductive technologies have been reported^6^^,^^7^; washed IUI can succeed but may leave seminal plasma remnants, trigger reactions, and require repeated attempts over time.^6^^,^^8^ IVF avoids maternal seminal plasma exposure while addressing age-related fertility concerns. Because the patient was 35 years old and desired early conception after severe systemic reactions, IVF was chosen over IUI and led to healthy childbirth after 2 cycles.^9^ To our knowledge, this is the first full case report of IVF-enabled live birth in HSPA, highlighting IVF as both a reproductive strategy and a preventive immunologic strategy. As assisted reproductive technologies become more accessible, IVF should be prioritized for women with severe seminal plasma allergy.

## Disclosure statement

This research did not receive any specific grant from funding agencies in the public, commercial, or not-for-profit sectors.

Disclosure of potential conflict of interest: The authors declare that they have no relevant conflicts of interest.
